# Aids to the Analysis of Food and Drugs

**Published:** 1896-09

**Authors:** 


**Aids to the Analysis of Food and Drugs.** By T. H. Pearmain and C. G. Moor. Pp. 160. London: Baillière, Tindall & Cox. [1895.]

The preface of this work contains the following sentences, which deserve the careful consideration of those who advocate the extension of the already unwieldy medical curriculum:—

"This work is not intended to be used as a cram-book for examinational purposes. We cannot emphasise too strongly the fact that food analysis is not to be taught in a few weeks, as is frequently attempted in the interest of public health students. A competent knowledge of the analysis of food and drugs is only to be attained by some years of active practical laboratory work." With this before us it becomes interesting to discover how such a mass of work can be adequately described in 140 small pages, the whole of which might be committed to memory by a fairly diligent student within the shorter time mentioned.

Now on endeavouring to put the processes as described into practice, it becomes apparent that, in the effort to condense as much as possible, explanatory details have been omitted to such an extent as to render the directions frequently unintelligible to the student, and occasionally entirely misleading. For instance, the very important process of butter analysis known as the Reichert-Meissl method modified by Wollny, who, by the way, is not mentioned, and the method for the detection of boric acid, both suffer so severely from the omission of necessary detail as to be quite incapable of yielding useful results; whilst it is hardly fair to lead the student to expect a satisfactory milk residue after treating the weighed quantity for three hours *on* and three hours *in* the water-bath. (The italics are the authors'.) And yet these descriptions are generally so free from direct misstatement that if transferred bodily to a written examination paper they would no doubt pass muster.

In fact, had the authors set out with the explanation that, in view of the heterogeneous jumble of information required of the public health student, there was urgent need of an undisguised cram-book which should provide in the most condensed form such descriptions of processes for the analysis of foods and drugs as would satisfy the examiner, they would have commanded our entire sympathy. For this is certainly one of the best examples of this much-abused class, the extent and general accuracy of the information conveyed in so small a compass being quite remarkable. Indeed the authors have interpreted the expression "Food and Drugs" in the most liberal manner, and have included articles upon soaps and urine. Altogether the book is one that can be strongly recommended to the examinee, whilst it is hardly likely to be of much service to the practical analyst.

